# Biodegradation Potential and Putative Catabolic Genes of Culturable Bacteria from an Alpine Deciduous Forest Site

**DOI:** 10.3390/microorganisms9091920

**Published:** 2021-09-10

**Authors:** Caroline Poyntner, Andrea Kutzner, Rosa Margesin

**Affiliations:** Department of Microbiology, University of Innsbruck, Technikerstrasse 25, 6020 Innsbruck, Austria; caroline.poyntner@uibk.ac.at (C.P.); andrea.kutzner@student.uibk.ac.at (A.K.)

**Keywords:** *Pseudomonas*, *Rhodococcus*, *Collimonas*, biodegradation, phenol, catechol, bisphenol A, lignin sulfonic acid, catabolic genes, low temperature

## Abstract

Microbiota from Alpine forest soils are key players in carbon cycling, which can be greatly affected by climate change. The aim of this study was to evaluate the degradation potential of culturable bacterial strains isolated from an alpine deciduous forest site. Fifty-five strains were studied with regard to their phylogenetic position, growth temperature range and degradation potential for organic compounds (microtiter scale screening for lignin sulfonic acid, catechol, phenol, bisphenol A) at low (5 °C) and moderate (20 °C) temperature. Additionally, the presence of putative catabolic genes (catechol-1,2-dioxygenase, multicomponent phenol hydroxylase, protocatechuate-3,4-dioxygenase) involved in the degradation of these organic compounds was determined through PCR. The results show the importance of the *Proteobacteria* phylum as its representatives did show good capabilities for biodegradation and good growth at −5 °C. Overall, 82% of strains were able to use at least one of the tested organic compounds as their sole carbon source. The presence of putative catabolic genes could be shown over a broad range of strains and in relation to their degradation abilities. Subsequently performed gene sequencing indicated horizontal gene transfer for catechol-1,2-dioxygenase and protocatechuate-3,4-dioxygenase. The results show the great benefit of combining molecular and culture-based techniques.

## 1. Introduction

Alpine forests are vulnerable ecosystems expected to be affected by the climate crisis through increasing temperatures, hydrogeological and drought events [1,2]. Important players are soil microbes, which control carbon cycling through carbon sequestration and carbon fluxes, e.g., those of carbon dioxide or methane [3,4,5]. It is also indicated that better understanding of microbial soil processes could improve Earth system models and predictions of climate change [6]. Positive feedback loops through climate warming are likely to have an effect on soils as carbon sinks which, in turn, results in increased carbon fluxes into the atmosphere and soil carbon loss [7]. The recent IPCC report states with high confidence that “the magnitude of feedbacks between climate change and the carbon cycle becomes larger but also more uncertain in high CO_2_ emissions scenarios” [8]. Therefore, understanding of these feedback processes is important. The microbial impact is often underestimated, particularly in cold and temperate soils [3]. 

Carbon cycling in soil is regulated by microbes through extracellular enzyme production, which degrade polymeric organic substances such as plant materials based on cellulose, hemicellulose, or lignin. External factors, e.g., temperature change, can directly or indirectly affect these enzymatic activities [9]. Bacteria are reported to be able to degrade lignin under aerobic conditions using extracellular laccases or peroxidases [10]. The depolymerized materials mostly containing phenol can be further degraded through hydroxylation via single or multicomponent hydroxylase (MPH). In the next step, ring cleaving enzymes such as catechol-1,2-dioxygenase (C1,2D) [11] catalyze the degradation of catechol via the ortho-cleavage pathway. Catechol is a central intermediate not only in the degradation of aromatic compounds from plant-based material but also in pollutant degradation [12]. For example, *Proteobacteria* are reported to degrade phenanthrene to catechol, ultimately leading to the tricarboxylic acid cycle [13]. Another known enzyme, protocatechuate-3,4-dioxygenase (P3,4D), is reported to oxidize phenolic compounds derived from lignin [14]. Additionally, it was shown that genes encoding for protocatechuate are upregulated in bacteria able to degrade the plasticizer and endocrine-disrupting chemical bisphenol A (BPA) [15]. *Stenotrophomonas maltophilia* cell extracts showed P3,4D activity when incubated with 4-hydroxybenzoic acid, a BPA degradation side product [16,17]. This shows that these bacterial enzymes are also important players for degradation of anthropogenic pollutants introduced into the environment.

To understand these degradation processes in Alpine forest soils, several previous studies were conducted in the Italian Alps at an elevation gradient [18,19,20,21,22] using culture-dependent and culture-independent methods [21,22]. Isolates from a submontane deciduous forest site, M, showed a high number of prokaryotic strains able to grow over a broad range of temperature, including low temperatures such as 0 °C. Additionally, the functional characterization of microbial communities demonstrated the presence of genes involved in the degradation of lignin and aromatic compounds [21]. 

Based on these previous results, the objective of this study was to evaluate the degradation potential of selected culturable bacterial strains. Fifty-five strains were studied by using both culture-based screening at a microtiter-scale and molecular-based detection of the presence of representative catabolic genes (C1,2D, MPH, P3,4D) involved in the degradation of organic model compounds. Four organic compounds were chosen for the degradation screening: (i) lignin sulfonic acid (LSS), (ii) catechol, (iii) phenol and (iv) BPA. LSS was chosen as a model compound for lignin. Catechol and phenol are intermediates from the lignin degradation process, but also structural compounds of various polymeric organic substrates. Further, it was hypothesized that the strains’ enzymatic toolkit designed to degrade lignin and phenols might also be useful in the degradation of BPA, as was reported in fungi [23]. The culture-based screening was performed at both low (5 °C) and moderate temperatures (20 °C) to detect degradation potential changes at different temperatures. The combination of molecular and culture-based techniques resulted in an interesting set of strains with degradation capabilities at different temperatures. These strains could also be applicable for bioremediation. 

## 2. Materials and Methods

### 2.1. Sampling and Isolation of Bacteria

The sampling site (deciduous Alpine forest) has been described in detail by França et al. (2016) [22]. Briefly, submontane site M (N 46°25′36.8″, E 11°17′48.6″) is located 8 km south of Bozen/Bolzano on a small peak, Kleiner Priol, at an altitude of 545–570 m above sea level. The pedogenic substratum consists of rhyolite (quartz-porphyry) and the soil was classified as dystric cambisol (FAO). The site consists of mixed deciduous forest, dominated by *Quercus pubescens*, *Q. robur*, *Fraxinus ornus*, *Pinus sylvestris* and *Ostrya carpinifolia*. The climate is mild continental with sub-Mediterranean influences, with an annual precipitation of 900 mm, a mean annual air temperature of 11.0 °C, a mean annual soil temperature of 9.8 °C, a minimum annual soil temperature of 6.5 °C and a maximum annual soil temperature of 13.5 °C [22].

Soil samples were collected from this site in late autumn (15 November 2014). Immediately after sampling, soil samples were transported to the laboratory in cooled boxes, sieved (<2 mm) and immediately analyzed for culturable microorganisms. The determination of soil characteristics [19] demonstrated a soil pH of 4.5. Contents of humus, total organic carbon and total N were 17%, 10% and 0.5%, respectively. The C/N ratio was 20. The 55 bacterial strains described in this study were isolated on R2A agar supplemented with cycloheximide (400 µg/mL) at 0 °C [22] and stored at −80 °C in R2A broth supplemented with 15% (*w/v*) glycerol.

### 2.2. Identification and Phylogenetic Analysis of Culturable Bacteria

Bacterial genomic DNAs were extracted by microwave lysis [24] and their quality and quantity were measured with a Nanodrop instrument (Thermo Fisher Scientific Inc., Waltham, MA, USA). Primers 27F and 1541R (Table S1, [25]) were used for partial 16S rRNA gene amplification. Each 50 µL PCR reaction contained 5 µL of PCR buffer (10×) (BIORON GmbH, Rauhweiden, Germany), 1 µL MgCl_2_ (100 mM) (BIORON GmbH, Rauhweiden, Germany), 1 µL dNTPs solution (10 mM each) (Sigma-Aldrich, St. Louis, MO USA), 2 µL each of forward and reverse primers (10 µM) (Eurofins Genomics, Luxembourg), 0.5 µL of Taq polymerase solution (5 U/µL) (BIORON GmbH, Rauhweiden, Germany), 4 µL of DNA extract, and 34.5 µL H_2_O. After the PCR (cycling program see Table S1), 16S rRNA PCR gene products were visualized on an ethidium bromide-stained agarose gel, purified using a GENEJET PCR purification kit (Thermo Fisher Scientific Inc., Waltham, USA) according to the manufacturer’s instructions, quality controlled on a Nanodrop instrument (Thermo Fisher Scientific Inc., Waltham, MA, USA) and Sanger sequenced (Microsynth AG, Balgach, Switzerland).

The sequences were manually edited using the software MEGA X [26]. The nearest phylogenetic neighbors of each strain were determined using the EzBioCloud 16S Database [27]. The sequences were clustered into operational taxonomic units (OTUs) at 99% identity using the average neighbor algorithm with the software Mothur v.1.39.3 [28]. All 16S rRNA gene sequences were deposited in GenBank NCBI [29] under the accession numbers shown in Table S2. 

A phylogenetic tree based on the closest type strains of the OTU sequences was created using Mega X [26]. The Tamura 3-parameter model [30] was applied. For the bootstrap consensus tree, 2000 replicates were used based on the neighbor joining method. A discrete gamma distribution (5 categories (+G, parameter = 0.7454)) and partial deletion option were applied. 

### 2.3. Growth Temperature Range of Bacterial Strains

Suspensions of bacterial cells (pre-grown on R2A) in 0.9% NaCl were used to inoculate R2A agar plates that were incubated at −5, 0, 5, 10, 15, 20, 25, 30, 35 and 40 °C, using two replicates per strain and temperatures. Growth was monitored over an incubation time of 7–28 days.

### 2.4. Screening for Utilization of Organic Compounds as Sole Carbon Source 

This screening was performed in microtiter plates (flat bottom, 96 wells), using 200 µL mineral medium (MM; [31]) per well. Each well received 100 µL of MM and 50 µL of a stock solution of various carbon sources (LSS, 8999, Carl Roth Gmbh, Karlsruhe, Germany; catechol, C-9510, Sigma-Aldrich, St. Louis, MO, USA; phenol; BPA, A10324, Sigma-Aldrich, St. Louis, MO, USA) diluted in MM to give final concentrations of 2 g/L and 5 g/L (LSS) or 0.2 g/L and 0.5 g/L (catechol, phenol, BPA). Then, the medium containing one of the carbon sources was inoculated with 50 µL of a suspension of bacterial cells (pre-grown in R2A broth and washed twice) in MM. Inoculated microtiter plates without carbon source and sterile plates were used as negative controls. In the case of BPA, growth (co-metabolic utilization) was additionally evaluated in MM supplemented with yeast extract (YE; 1 g/L). Controls contained MM with YE, and growth in (MM + YE + BPA) was corrected for growth in (MM + YE). 

Microtiter plates were wrapped in plastic bags, incubated at 5 °C and at 20 °C, and growth was determined regularly over a period of 14–21 days using a microplate reader. Growth was considered positive at an OD_600_ value > 0.1. The OD_600_ was measured spectrophotometrically (Hitachi High Tech, Tokyo, Japan). Three replicates were prepared per strain, compound, and temperature. 

### 2.5. PCR-Based Detection of Putative Catabolic Genes

The extracted DNA of 55 strains was further used to screen for putative catabolic genes based on previous published studies: genes encoding for P3,4D [32], C1,2D [33] and MPH [33]. The primer sequences and PCR conditions are described in Table S1. Two negative controls were used: (i) without a DNA template and (ii) with an *E. coli* DNA template. Positive controls contained DNA templates of strains with known presence of the targeted genes: *Pseudomonas putida* DSM3931 for C1,2D [34], *Parakburkholderia aromaticivorans* AR20-38 for MPH and P3,4D [35]. For the screening of all 55 strains, 25 µL of reaction mixtures contained 5 µL PCR of buffer B (10×) (Nippon Genetics Europe GmbH, Düren, Germany), 0.5 µL of dNTPs solution (10 mM each) (Sigma-Aldrich, St. Louis, MO, USA), 1 µL each of forward and reverse primers (10 µM) (Eurofins Genomics, Luxembourg), 0.5 µL of Fast Gene Taq polymerase (5 U/µL) (Nippon Genetics Europe, Düren Germany), 2 µL of DNA extract, 0.5 µL of Bovine Serum Albumin solution (20 mg/mL) (New England Biolabs, Ipswich, MA, USA) and 14.5 µL of H_2_O. After the PCR reaction, putative PCR products were visualized as mentioned above (Section 2.2). Twenty reactions were selected for sequencing and were rerun on a 50 µL scale. Purification, quality check and sequencing were performed as mentioned above (Section 2.2). All sequences were deposited in GenBank NCBI [29] under the accession numbers shown in Table S3.

A phylogenetic tree of the resulting sequences was produced with Mega X [26]. The Maximum Likelihood method and Tamura 3-parameter models were used [30]. Two thousand replicates were used for the bootstrap consensus tree. Closely related sequences from blastx and blastn (https://blast.ncbi.nlm.nih.gov/, access on 10 August 2021) and type gene sequences from published references [31,32] were included. All positions with less than 95% site coverage were eliminated.

### 2.6. Phenol Degradation

Strain AM0-06 with the presence of the gene for MPH was tested for phenol degradation at 5 °C and 20 °C in liquid culture in 100-mL Erlenmeyer flasks containing 20 mL of MM with phenol concentrations of 0.2, 0.5, 0.75 and 1.0 g/L. Growth (OD_600_) and the residual phenol content [36,37] were monitored regularly. Phenol concentration was determined in culture supernatants that were filtered (0.2 µm, Minisart RC4 17821, Sartorius AG, Göttingen, Germany) after centrifugation. High-performance liquid chromatography (HPLC) was carried out by using a RP-18 column (5 µm × 100 mm, Lichrospher, Merck, Darmstadt, Germany), with UV detection at 220 nm (SPD-20A, Shimadzu Scientfic, Columbia, MD, USA) and an eluent flow of 0.5 mL/min. The elution time for phenol was approx. 7.5 min. The phenol calibration curve was prepared in MM.

### 2.7. Catechol-1,2-dioxygenase Activity

Strain AM0-06 with the presence of the gene encoding for C1,2D was selected for the determination of this enzyme activity. The strain was grown in triplicate in liquid cultures at 5 °C and 20 °C in 100-mL Erlenmeyer flasks in the complex R2A medium (constitutively expressed activity) and in MM with phenol (0.2 g/L and 0.5 g/L) (induced activity). After centrifugation, cell pellets were washed twice and suspended in 0.9% NaCl; the OD_600_ value of all cell suspensions was adjusted to 1.5. By using these standardized cell suspensions, it was possible to compare the activities of the strain grown under various conditions. The formation of *cis*,*cis*-muconic acid from catechol was determined at 260 nm as described [36,38].

## 3. Results

### 3.1. Culturable Bacterial Diversity

**Figure 1:**
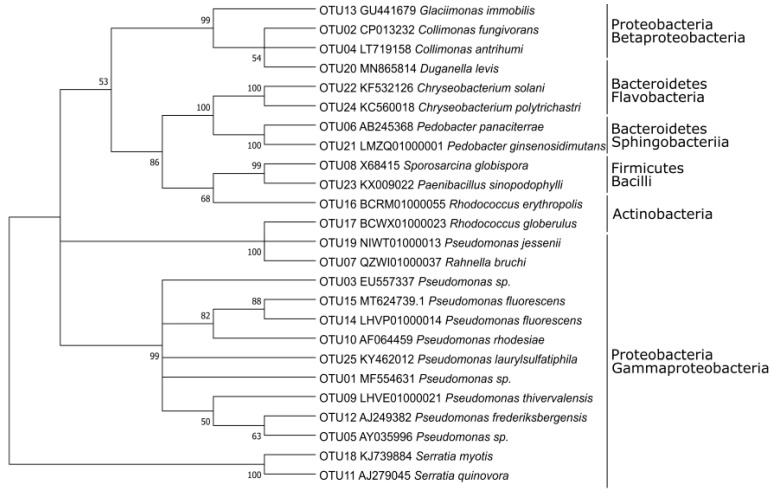
Phylogenetic relationship of the operational taxonomic units (OTUs) based on 16S rRNA gene sequences of the isolated strains and their reference species.

The phylogenetic relationship between the studied strains is shown in Figure 1. The majority of the studied 55 strains (45, 82%) belonged to the phylum *Proteobacteria*, with the predominance of the class *Gammaproteobacteria* (two-thirds of *Proteobacteria*) over *Betaproteobacteria* (one-third of *Proteobacteria*), while *Alphaproteobacteria* were not present (Figure 1, Table 1). The genus *Pseudomonas* (26 of 30 strains) dominated among *Gammaproteobacteria*, and *Betaproteobacteria* were dominated by the genus *Collimonas* (14 of 15 strains). Only 11% (six strains) were represented by *Bacteroidetes* (genera *Pedobacter, Chryseobacterium, Duganella*). The remaining four strains were related to phyla *Actinobacteria* and *Firmicutes* (two strains each) (Figure 1, Table 1). Thus, the fraction of Gram-positive bacteria (7%) was very small compared to that of Gram-negative bacteria (93%). 

The 55 strains were grouped into 25 OTUs at 99% identity (Table 1). Sixteen OTUs were clustered *Proteobacteria*, thereof 13 in *Gammaproteobacteria* and three in *Betaproteobacteria*. Two OTUs predominated: OTU01 comprised 13 strains belonging to the genus *Pseudomonas* (order *Pseudomonadaceae, Gammaproteobacteria);* OTU02 clustered twelve strains of order *Collimonas* (*Oxalobacteraceae, Betaproteobacteria*). All the other OTUs comprised four or less strains (Table S3). The phylogenetic tree (Figure 1) shows that the different classes cluster well together, and that *Gammaproteobacteria* predominate. 

### 3.2. Growth Temperature Range

**Figure 2:**
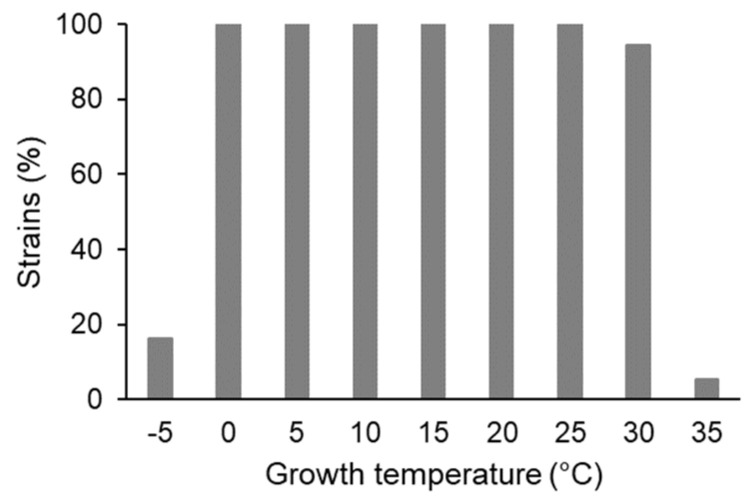
Growth temperature range of the tested bacterial strains (55 = 100%).

All 55 tested strains could grow at temperatures ranging from 0 to 25 °C; 16% of them (nine strains) could even grow at −5 °C (Figure 2). Among the latter, the genus *Pseudomonas* dominated (seven of nine strains; distributed in six different OTUs, three of them in OTU5); the other two strains belonged to the phyla *Bacteroidetes* (*Chryseobacterium*, one strain) and *Firmicutes* (*Sporosarcina*, one strain). 

The majority (52 strains, 94.5%) could grow from 0 to 30 °C; among them were all representatives of OTU01 (*Pseudomonas*, 13 strains) and OTU02 (*Collimonas*, twelve strains). The three strains able to grow at 35 °C belonged to genera *Serratia* (two strains, OTU11 and OTU18) and *Rhodococcus* (one strain, OTU16); none of these strains could grow at 40 °C (Table S2). 

### 3.3. Screening for Utilization of Organic Compounds as Sole Carbon Sources 

The 55 culturable strains were screened for their ability to utilize two concentrations of LSS, catechol, phenol, and/or BPA as sole carbon sources at both 5 °C and 20 °C. The detailed data are reported in Table S2. 

LSS served as a carbon source for a large fraction of the tested strains (Table 2 and Table S2). The higher concentration tested (5 g/L) was utilized by almost two-thirds (35 strains, 63.6%) at 20 °C and by almost half of the tested 55 strains at 5 °C (25 strains, 45.5%). Twenty-two strains (40%) utilized this LSS concentration at both test temperatures, while just three and 13 strains utilized it only at 5 °C or 20 °C, respectively. The lower LSS concentration (2 g/L) resulted in a significantly lower amount of positive strains at both temperatures (five strains, 9.1%) than the higher concentration; four strains utilized this concentration only at 5 °C or 20 °C. Remarkable growth (OD_600_ > 1) was noted for two strains (AM0-90 and AM0-92, belonging to the genus *Pseudomonas*, OTU03) at both concentrations and both test temperatures, and for strain AM0-28 (*Rhodococcus*, OTU16) at both concentrations and 20 °C. 

The number of strains able to degrade catechol (Table 2 and Table S2) increased with its concentration and temperature. Eleven (20%) and 19 strains (34.5%), respectively, were able to utilize the lowest (0.2 g/L) or highest catechol concentration (0.5 g/L) tested at both 5 °C and 20 °C. Among these strains, six utilized both concentrations at both temperatures. They all belonged to the genus *Pseudomonas* and were distributed in five OTUs; the highest amounts of biomass were produced by strains AM0-90 and AM0-92. Two strains were able to utilize catechol at 5 °C, but not at 20 °C: AM0-51 (*Pseudomonas*, OTU01) degraded the lowest concentration and AM0-02 (*Rhodococcus*, OTU17) degraded the highest concentration.

Phenol was degraded at both concentrations tested (0.2 and 0.5 g/L) by ten strains (18.2%) at 20 °C (Table 2 and Table S2). At 5 °C, eight of these strains (14.5% of 55) still utilized the lowest concentration, while only half of them (four strains) could degrade the highest amount of phenol. None of the 55 strains utilized phenol, independent of the concentration, only at 5 °C. Two and six strains degraded 0.2 and 0.5 g/L, respectively, only at 20 °C. The four strains degrading both phenol concentrations both at 5 °C and 20 °C belonged to the genera *Pseudomonas* (OTU03 (two strains, AM0-90, AM0-92) and OTU09 (one strain, AM0-06)) and *Rhodococcus* (OTU16, AM0-28). 

BPA was only utilized by a very low fraction of 55 strains as the sole carbon source (Table 2 and Table S2), independent of either concentration (0.2 and 0.5 g/L) or temperature. Only two strains (AM0-90 and AM0-92, *Pseudomonas*) could grow with both concentrations as the sole carbon source at 5 °C and 20 °C. Two other strains utilized only the lowest BPA concentration at 20 °C; they both belonged to the genus *Serratia* but to different OTUs (AM0-14, OTU18; AM0-19, OTU11). The highest amount of biomass (OD_600_ > 1) was produced by strain AM0-90 at 20 °C. None of the strains degraded BPA only at 5 °C. 

The evaluation of co-metabolic degradation of BPA in the presence of YE resulted in an increased number of positive strains (Table 2 and Table S2). Four and eight strains utilized the lowest (0.2 g/L) or highest BPA concentration (0.5 g/L) at 20 °C, respectively. The number of positive strains at 5 °C decreased with increasing the BPA amount. Strain AM0-62 (*Collimonas*, OTU02) was the only strain able to utilize both BPA concentrations only at 5 °C. The three strains degrading 0.2 g/L BPA at both 5 °C and 20 °C (AM0-18, AM0-30, AM0-81) belonged to the same cluster (*Collimonas*, OTU02). *Collimonas* representatives produced the highest amount of biomass (OD_600_ < 0.4). 

### 3.4. PCR-Based Detection of Biodegradation Related Genes 

The 55 strains used were screened for the presence of known genes related to the enzymes involved in the biodegradation of the tested compounds: MPH, C1,2D, and P3,4D. The PCR screening approach was successful over a wide range of strains and provided an overview of the putative catabolic genes. Results could be related to the culture-based screening results (Table S2). Seven strains showed a PCR product for MPH, 22 for P3,4D and 25 for C1,2D (Table S2). Only two strains, AM0-71 (*Pseudomonas* sp., OTU01) and AM0-06 (*Pseudomonas* sp., OTU09), showed PCR products for all three genes. Due to the good degradation performance of strain AM0-06 in the screening, its capability for phenol degradation (see Section 3.5) and C1,2D activity (see Section 3.6) were studied further. Eight strains (AM0-14, AM0-19, AM0-28, AM0-58, AM0-64, AM0-65, AM0-81, AM0-85), which showed biodegradation abilities in the microtiter scale screening, did not show the right size of PCR products or no PCR products. This might be a result of primer design and PCR conditions, which were designated for a broad range of species. 

Twenty PCR products were selected for sequencing based on the growth (OD > 0.25) of the corresponding strains in the presence of the tested organic compounds as the sole carbon sources (Table S2). Sequence similarities to published C1,2D, MPH and P3,4D gene sequences were evaluated with Blastx and Blastn (https://blast.ncbi.nlm.nih.gov/, access on 10 August 2021). MPH gene sequences showed 90–96% identity, the gene sequences of P3,4D 94–100% and the gene sequences of C1,2D 93–100%, respectively. 

**Figure 3:**
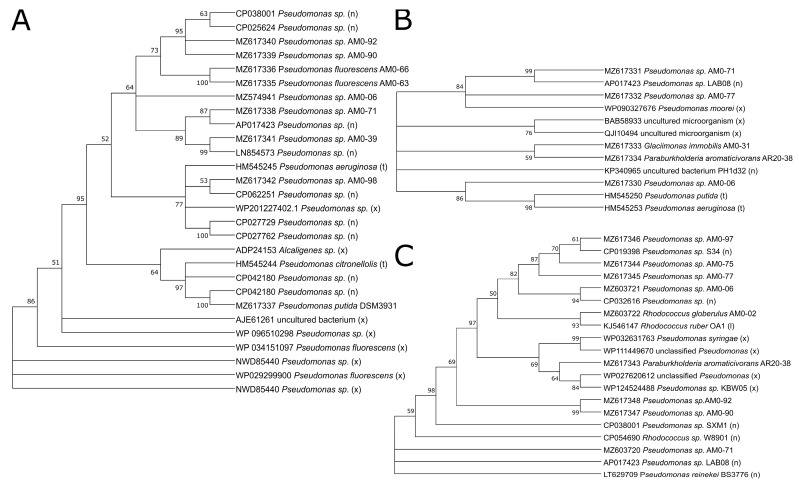
Phylogenetic tree of the studied (**A**) catechol-1,2-dioxygenase (**B**) multicomponent phenol hydroxylase and (**C**) protocatechuate-3,4-dioxygenase gene sequences. Strain numbers and accession numbers from GenBank are indicated. (n) and (x) indicate closest sequences based on NCBI blastn and blastx. (t) and (l) indicate published sequences by [32,33]. Bootstrap probabilities are shown at the branch nodes.

The gene sequences encoding for C1,2D, present in 25 of the 55 studied strains, were predominantly found in the representatives of *Pseudomonas* sp. (18 strains, distributed in 3 OTUs) and *Collimonas* (5 strains, OTU02). The sequences in the phylogenetic tree of the C1,2D putative gene sequences are related to known C1,2D genes found in NCBI (marked with (x) and (n) in Figure 3A). The published sequence of *Pseudomonas citronellolis* [33] clustered well within the sequence results obtained in our study, especially with the sequence from *Pseudomonas putida* DSM3931 used as a control. The topology of the C1,2D sequences-based tree (Figure 3A) differed from the 16S rRNA gene-based tree (Figure 1). While the C1,2D sequences of strains AM0-90 and AM0-92 are closely related and both strains belong to the same OTU, strains AM0-66 (OTU15), AM0-63 (OTU14), AM0-39 (OTU03) and AM0-71 (OTU01) are clustered together in the C1,2D sequences-based tree (Figure 3A) but their OTUs are not closely related in the phylogenetic tree (Figure 1). This might indicate a horizontal gene transfer between the different *Pseudomonas* representatives. Interestingly, the putative C1,2D gene sequence of strain AM0-98 is distant from that of strain AM0-71, although both strains are grouped in OTU01. 

The gene sequences encoding for MPH, present in seven of the 55 studied strains, were predominantly found in the representatives of *Pseudomonas* sp. (five strains). The MPH gene sequences, which are a group in the same OTU (OTU01) (strains AM0-71 and AM0-77, Figure 1), are closely related (Figure 3B) and are similarly distributed as in the phylogenetic tree (Figure 1). Sequences from the genus *Pseudomonas* are distributed in two clusters: either those closely related to *Pseudomonas moorei* (AM0-71 and AM0-77) or to *Pseudomonas putida* and *Pseudomonas aeruginosa* (AM0-06). The putative MPH gene sequence of *Glaciimonas immobilis* (strain AM0-31) shows less relation to the sequences of the other strains studied. 

The phylogenetic tree of putative P3,4D genes (Figure 3C) found in 22 strains is similar to the relationships shown in Figure 3A. Strains AM0-90 and AM0-92 are closely related and cluster in the same OTU03. Strains AM0-75 and AM0-77 have closely related sequences, while the third representative of their OTU (01), AM0-71, is not closely related. The sequence reported by Li et al. [32] is closely related to the sequence from strain AM0-02 and belongs to the same genus (*Rhodococcus*). 

### 3.5. Phenol Degradation

**Figure 4:**
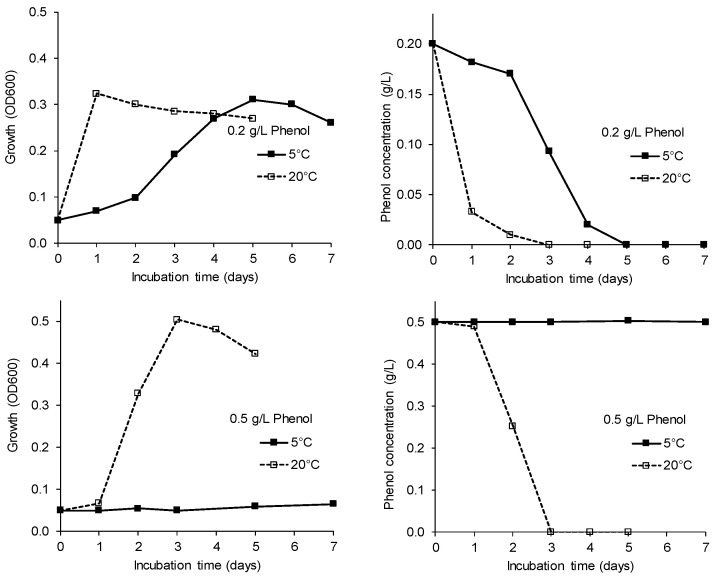
Effect of temperature and phenol concentration (top panel: 0.2 g/L; bottom panel: 0.5 g/L) on growth (left panels) and phenol degradation (right panels) by strain *Pseudomonas* sp. AM0-06 (mean values of three replicates, SDs were ≤10%).

The phenol degradation potential of strain AM0-06 (*Pseudomonas* sp.) was tested in the liquid culture at 5 °C and 20 °C. A concentration of 0.2 g/L phenol was almost fully degraded after one day at 20 °C, whereas complete degradation at 5 °C was detected after five days (Figure 4). The higher phenol concentration (0.5 g/L) was degraded at 20 °C within three days, while no degradation could be observed at 5 °C. Concentrations of 0.75 and 1.0 g/L phenol were not utilized at any of the tested temperatures within 15 days. 

### 3.6. Catechol-1,2-dioxygenase Activity

The C1,2D activity is involved in the second step of phenol degradation and catalyzes the degradation of catechol via the ortho-cleavage pathway. Strain AM0-06 demonstrated both the constitutively expressed and induced C1,2D activity. However, activities were significantly higher in the cells grown with phenol as the sole carbon source (induced; 32-36 U) compared to the complex medium (constitutive; 4-6 U). 

## 4. Discussion

The phylum *Proteobacteria* is reported as the most abundant in global soil microbiota analysis [39]. This was also previously demonstrated at the Alpine submontane deciduous forest site, M, through culture-based data [22] and amplicon sequencing data [21]. In the study reported here, 55 strains from that site were tested on their phylogenetic position, growth temperature range, degradation potential for organic compounds and the presence of putative catabolic genes related to the organic compounds tested. The majority of these strains (82%) belonged to the phylum *Proteobacteria*. Among them, the genus *Pseudomonas* (*Gammaproteobacteria*) predominated (26 strains) and also built the largest OTU (OTU01, 13 strains). The second predominating genus with 14 representatives was *Collimonas* (*Gammaprotebacteria*), the majority of them were clustered in the second largest OTU (OTU02, twelve strains). Both genera are known for various cold-adapted representatives [22,40,41,42], the production of cold-adapted enzymes and low-temperature degradation of organic compounds [42,43]. Among the 55 strains, no *Alphaproteobacteria* and only a very low fraction of *Bacteroidetes* (six strains) and Gram-positive representatives (four strains) were detected. The latter can be related to lower competitiveness compared to Gram-negative bacteria [42]. The low biodiversity might be a result of site-specific soil conditions, characterized by lower contents of soil organic matter and soil nutrients compared to a previously studied Alpine coniferous forest site [42]. 

In adaptation to the site-specific climate conditions, the majority of the strains (94.5%) were able to grow well over a broad temperature range from 0 °C to 30 °C, among them all representatives of genera *Pseudomonas* and *Collimonas*. Remarkably, seven of the 13 *Pseudomonas* representatives were able to grow at −5 °C. Only two other strains, *Chryseobacterium* and *Sporosarcina*, could grow at this low temperature. Microbial growth and activity at subzero temperatures have been reported for a wide variety of microorganisms from cold ecosystems [44].

The previous functional characterization of microbial communities from the source of the studied strains demonstrated the presence of genes involved in the degradation of lignin and aromatic compounds [21]. The C/N ratio of 20 in the soil samples at the studied forest site is favorable for biodegradation processes. Therefore, the culturable strains tested in this study were evaluated for their ability to degrade two concentrations of a number of organic compounds (LSS, catechol, phenol, BPA), both at low (5 °C) and moderate (20 °C) temperature. While LSS, catechol and phenol were chosen as model compounds for lignin degradation, it was hypothesized that the bacterial ligninolytic enzymes might be good tools to degrade BPA, an endocrine-disrupting pollutant. This was the case for three strains (AM0-19, AM0-90, AM0-92), which showed good performance in LSS, phenol, catechol and BPA degradation. Nevertheless, no general correlation between the degradation of lignin-derived substances and BPA could be observed, which is in contrast to reported mechanisms in fungi [23]. 

The best performance in utilization of these compounds as sole carbon sources was observed with representatives of the genera *Pseudomonas* and *Rhodococcus*. In our study, two *Pseudomonas* sp. strains (AM0-90 and AM0-92) utilized both concentrations of all four compounds tested as sole carbon sources at 5 °C and 20 °C. They also produced high amounts of biomass compared to other strains (Table S2). Only *Pseudomonas* representatives were able to degrade both concentrations of catechol and BPA at both temperatures. Next to *Pseudomonas*, *Rhodococcus* sp. was able to degrade phenol at all tested conditions. This shows the remarkable degradation performance of the genera *Pseudomonas* and *Rhodococcus* in the environment. Low-temperature phenol degradation by strains of these two genera has been reported [45,46]. Species of both genera have been frequently isolated from contaminated soils and reported as efficient degraders of organic compounds [47,48,49]. 

The results of our study demonstrated the effect of compound concentration and temperature on biodegradation. Regarding LSS and catechol, the lower concentration (2 g/L and 0.2 g/L, respectively) was utilized by a lower fraction of strains compared to the higher concentration (5 g/L and 0.5 g/L, respectively) at both temperatures. The same trend was observed for BPA degradation in the presence of YE. By contrast, the number of strains able to degrade phenol decreased with increasing concentration (0.2 g/L vs. 0.5 g/L) at the lower temperature (5 °C) compared to 20 °C. This points to an inhibiting, toxic low-temperature effect of phenol, as reported earlier [45]. Phenol and phenolic compounds are known to be highly toxic to microorganisms [50]. 

The investigation of the presence of putative catabolic genes confirmed the degradation ability demonstrated in culture-based experiments. Two *Pseudomonas* strains, AM0-90 and AM0-92, showed the presence of the catabolic genes for C1,2D (indicative for catechol degradation) and PD3,4D. On a phylogenetic level (Figure 3), their putative gene sequences are closely related to each other. This is in line with their clustering in the same OTU (Figure 1) and the results obtained from the culture-based biodegradation screening (Table S2). However, despite the ability of these strains to degrade phenol, the MPH gene was not detected, which suggests biodegradation via a single component monooxygenase rather than MPH. Another possibility is the requirement of strain-specific primers to directly target the MPH gene cluster at a different location. MPH primers in this study targeted the α-subunit of the MPH amino acid sequence [33]. 

Protocatechuate is an intermediate in a number of catabolic pathways involving compounds with aromatic rings, such as lignin, phenol and BPA [15,51]. P3,4D PCR products were shown for six out of the seven *Pseudomonas* strains, which utilized BPA as either the sole carbon source or in the presence of yeast extract. Therefore, the P3,4D gene seems to be an important factor for BPA utilization within the genus *Pseudomonas*. Representatives of the genera *Collimonas* and *Serratia* were also able to grow in the presence of BPA but did not show PCR products for P3,4D. This might be a result of unspecific primers for the P3,4D genes of these genera; alternatively, they might use a different catabolic pathway to utilize BPA. 

The phylogenetic relationships of the different catabolic gene sequences evaluated in this study (Figure 3) indicate the possibility of a horizontal gene transfer for C1,2D and P3,4D, since the phylogenetic gene-based relationship differs from the phylogenetic 16S rRNA-based relationship (Figure 1). This has previously been reported [33,52]. 

## 5. Conclusions

In conclusion, the results obtained in our study demonstrate the successful use of PCR to detect potential catabolic genes and point to the usefulness of this method in combination with culture-based screening for the utilization of organic compounds as sole carbon sources to evaluate the biodegradation potential of a wide range of strains. The primers used in this study proved to be successful for many different strains. Strains of interest can then be studied further in detail for their degradation potential, such as the effect of temperature and concentration, as shown in this study for the strain *Pseudomonas* sp. AM0-06. This strain degraded phenol and catechol on a microtiter scale screening and possessed the genes for MPH and C1,2D, which are involved in phenol and catechol degradation. The evaluation of phenol degradation at two concentrations and temperatures in liquid culture and the quantification of C1,2D activity via photometry confirmed the efficient degradation ability of the strain and the use of C1,2D to degrade catechol, produced from phenol degradation, via the ortho-cleavage pathway. In addition, our study demonstrates the potential of bacterial strains isolated from Alpine forest sites for biodegradation of organic compounds at low (5 °C) and moderate temperatures (20 °C), which is desirable in the context of bioremediation under changing climate conditions. 

## Figures and Tables

**Table 1:** Identification of the 55 studied culturable bacterial strains (*n* = number of representatives).

Phylum (*n*)	Class (*n*)	OTUs per Class (*n*)	Genera (*n*)	OTUs per Genus (*n*)
*Actinobacteria* (2)		2	*Rhodococcus* (2)	2
*Firmicutes* (2)		2	*Sporosarcina* (1)	1
			*Paenibacillus* (1)	1
*Bacteroidetes* (6)		5	*Pedobacter* (3)	2
			*Chryseobacterium* (2)	1
			*Duganella* (1)	2
*Proteobacteria* (45)	*Gammaproteobacteria* (30)	13	*Pseudomonas* (26)	10
			*Rhanella* (2)	1
			*Serratia* (2)	2
	*Betaproteobacteria* (15)	3	*Collimonas* (14)	2
			*Glaciimonas* (1)	1

**Table 2:** Effect of temperature and concentration on the bacterial utilization of organic polymers as sole carbon source (100% = 55, LSS = lignin sulfonic acid; BPA = bisphenol A).

Compound	Conc. (g/L)	5 °C		20 °C		5 °C only		20 °C only		5 °C and 20 °C	
		*n*	%	*n*	%	*n*	%	*n*	%	*n*	%
LSS	2.0	9	16.4	9	16.4	4	7.3	4	7.3	5	9.1
	5.0	25	45.5	35	63.6	3	5.5	13	23.6	22	40.0
Catechol	0.2	12	21.8	31	56.4	1	1.8	20	36.4	11	20.0
	0.5	20	36.4	45	81.8	1	1.8	26	47.3	19	34.5
Phenol	0.2	8	14.5	10	18.2	0	0.0	2	3.6	8	14.5
	0.5	4	7.3	10	18.2	0	0.0	6	10.9	4	7.3
BPA	0.2	2	3.6	4	7.3	0	0.0	2	3.6	2	3.6
	0.5	2	3.6	2	3.6	0	0.0	0	0.0	2	3.6
BPA + YE	0.2	4	7.3	4	7.3	1	1.8	1	1.8	3	5.5
	0.5	5	9.1	8	14.5	5	9.1	8	14.5	0	0.0

## Data Availability

Gene sequences were deposited at NCBI (https://www.ncbi.nlm.nih.gov/genbank/). Accession numbers are listed in Supplementary Materials Tables S2 and S3.

## Supplementary Materials

The following tables are available online at https://www.mdpi.com/article/10.3390/microorganisms9091920/s1. Table S1. Primers and PCR conditions for partial 16S rRNA gene amplification and the detection of putative catabolic genes: catechol-1,2-dioxygenase (C1,2D); multicomponent phenol hydroxylase (MPH); protocatechuate-3,4-dioxygenase (P3,4D). Table S2. Identification of the 55 strains investigated, growth temperature range on R2A agar, effect of temperature (5 °C and 20 °C) on the utilization of organic compounds as sole carbon source, and presence of catabolic genes. Table S3. Accession numbers of sequenced genes Catechol-1,2-dioxygenase (C1,2D), multicomponent phenol hydroxylase (MPH) and protocatechuate-3,4-dioxygenase (P3,4D) deposited at Genbank NCBI (https://www.ncbi.nlm.nih.gov/genbank/).

## References

[1] Böhm R., Auer I., Brunetti M., Maugeri M., Nanni T., Schöner W. (2001). Regional temperature variability in the European Alps: 1760–1998 from homogenized instrumental time series. Int. J. Climatol..

[2] Ciccarelli N., von Hardenberg J., Provenzale A., Ronchi C., Vargiu A., Pelosini R. (2008). Climate variability in north-western Italy during the second half of the 20th century. Glob. Planet. Chang..

[3] Cavicchioli R., Ripple W.J., Timmis K.N., Azam F., Bakken L.R., Baylis M., Behrenfeld M.J., Boetius A., Boyd P.W., Classen A.T. (2019). Scientists’ warning to humanity: Microorganisms and climate change. Nat. Rev. Microbiol..

[4] Praeg N., Wagner A.O., Illmer P. (2017). Plant species, temperature, and bedrock affect net methane flux out of grassland and forest soils. Plant Soil.

[5] Bardgett R.D., Freeman C., Ostle N.J. (2008). Microbial contributions to climate change through carbon cycle feedbacks. ISME J..

[6] Wieder W.R., Bonan G.B., Allison S.D. (2013). Global soil carbon projections are improved by modelling microbial processes. Nat. Clim. Chang..

[7] Walker T.W.N., Kaiser C., Strasser F., Herbold C.W., Leblans N.I.W., Woebken D., Janssens I.A., Sigurdsson B.D., Richter A. (2018). Microbial temperature sensitivity and biomass change explain soil carbon loss with warming. Nat. Clim. Chang..

[8] Masson-Delmotte V., Zhai P., Pirani A., Connors S.L., Péan C., Berger S., Caud N., Chen Y., Goldfarb L., Gomis M.I. IPCC, 2021: Summary for Policymakers. Climate Change 2021: The Physical Science Basis.

[9] D’Alò F., Odriozola I., Baldrian P., Zucconi L., Ripa C., Cannone N., Malfasi F., Brancaleoni L., Onofri S. (2021). Microbial activity in alpine soils under climate change. Sci. Total. Environ..

[10] Bugg T.D., Ahmad M., Hardiman E.M., Singh R. (2011). The emerging role for bacteria in lignin degradation and bio-product formation. Curr. Opin. Biotechnol..

[11] Merimaa M., Heinaru E., Liivak M., Vedler E., Heinaru A. (2006). Grouping of phenol hydroxylase and catechol 2,3-dioxygenase genes among phenol- and p-cresol-degrading *Pseudomonas* species and biotypes. Arch. Microbiol..

[12] Rodríguez-Salazar J., Almeida-Juarez A.G., Ornelas-Ocampo K., Millán-López S., Raga-Carbajal E., Rodríguez-Mejía J.L., Muriel-Millán L.F., Godoy-Lozano E.E., Rivera-Gómez N., Rudiño-Piñera E. (2020). Characterization of a Novel Functional Trimeric Catechol 1,2-Dioxygenase From a *Pseudomonas stutzeri* Isolated From the Gulf of Mexico. Front. Microbiol..

[13] Bianco F., Race M., Papirio S., Esposito G. (2021). Phenanthrene biodegradation in a fed–batch reactor treating a spent sediment washing solution: Techno–economic implications for the recovery of ethanol as extracting agent. Chemosphere.

[14] Harayama S., Rekik M. (1989). Bacterial aromatic ring-cleavage enzymes are classified into two different gene families. J. Biol. Chem..

[15] Zhou N.A., Kjeldal H., Gough H.L., Nielsen J.L. (2015). Identification of Putative Genes Involved in Bisphenol A Degradation Using Differential Protein Abundance Analysis of *Sphingobium* sp. BiD32. Environ. Sci. Technol..

[16] Urszula G., Izabela G., Danuta W., Sylwia Ł. (2009). Isolation and characterization of a novel strain of *Stenotrophomonas maltophilia* possessing various dioxygenases for monocyclic hydrocarbon degradation. Braz. J. Microbiol..

[17] Cydzik-Kwiatkowska A., Zielińska M. (2018). Microbial composition of biofilm treating wastewater rich in bisphenol A. J. Environ. Sci. Health Part A.

[18] Siles J.A., Cajthaml T., Filipová A., Minerbi S., Margesin R. (2017). Altitudinal, seasonal and interannual shifts in microbial communities and chemical composition of soil organic matter in Alpine forest soils. Soil Biol. Biochem..

[19] Siles J.A., Cajthaml T., Minerbi S., Margesin R. (2016). Effect of altitude and season on microbial activity, abundance and community structure in Alpine forest soils. FEMS Microbiol. Ecol..

[20] Siles J.A., Cajthaml T., Frouz J., Margesin R. (2019). Assessment of soil microbial communities involved in cellulose utilization at two contrasting Alpine forest sites. Soil Biol. Biochem..

[21] Siles J.A., Margesin R. (2017). Seasonal soil microbial responses are limited to changes in functionality at two Alpine forest sites differing in altitude and vegetation. Sci. Rep..

[22] França L., Sannino C., Turchetti B., Buzzini P., Margesin R. (2016). Seasonal and altitudinal changes of culturable bacterial and yeast diversity in Alpine forest soils. Extremophiles.

[23] Gassara F., Brar S.K., Verma M., Tyagi R.D. (2013). Bisphenol A Degradation in Water by Ligninolytic Enzymes. Chemosphere.

[24] Sánchez-Hidalgo M., Pascual J., de la Cruz M., Martín J., Kath G.S., Sigmund J.M., Masurekar P., Vicente F., Genilloud O., Bills G.F. (2012). Prescreening bacterial colonies for bioactive molecules with Janus plates, a SBS standard double-faced microbial culturing system. Antonie Van Leeuwenhoek.

[25] Lane D., Stackebrandt E., Goodfellow M. (1991). 16S/23S rRNA sequencing. Nucleic Acid Techniques in Bacterial Systematics.

[26] Kumar S., Stecher G., Li M., Knyaz C., Tamura K. (2018). MEGA X: Molecular Evolutionary Genetics Analysis across Computing Platforms. Mol. Biol. Evol..

[27] Yoon S.-H., Ha S.-M., Kwon S., Lim J., Kim Y., Seo H., Chun J. (2017). Introducing EzBioCloud: A taxonomically united database of 16S rRNA gene sequences and whole-genome assemblies. Int. J. Syst. Evol. Microbiol..

[28] Schloss P.D., Westcott S.L., Ryabin T., Hall J.R., Hartmann M., Hollister E.B., Lesniewski R.A., Oakley B.B., Parks D.H., Robinson C.J. (2009). Introducing mothur: Open-Source, Platform-Independent, Community-Supported Software for Describing and Comparing Microbial Communities. Appl. Environ. Microbiol..

[29] NCBI (2018). Resource Coordinators Database resources of the National Center for Biotechnology Information. Nucleic Acids Res..

[30] Tamura K. (1992). Estimation of the number of nucleotide substitutions when there are strong transition-transversion and G + C-content biases. Mol. Biol. Evol..

[31] Margesin R., Schinner F. (1997). Bioremediation of diesel-oil-contaminated alpine soils at low temperatures. Appl. Microbiol. Biotechnol..

[32] Li C., Zhang C., Song G., Liu H., Sheng G., Ding Z., Wang Z., Sun Y., Xu Y., Chen J. (2016). Characterization of a protocatechuate catabolic gene cluster in *Rhodococcus* ruber OA1 involved in naphthalene degradation. Ann. Microbiol..

[33] Tuan N.N., Hsieh H.-C., Lin Y.-W., Huang S.-L. (2011). Analysis of bacterial degradation pathways for long-chain alkylphenols involving phenol hydroxylase, alkylphenol monooxygenase and catechol dioxygenase genes. Bioresour. Technol..

[34] Nakai C., Kagamiyama H., Nozaki M., Nakazawa T., Inouye S., Ebina Y., Nakazawa A. (1983). Complete nucleotide sequence of the metapyrocatechase gene on the TOI plasmid of *Pseudomonas putida* mt-2. J. Biol. Chem..

[35] Poyntner C., Zhang D., Margesin R. (2020). Draft Genome Sequence of the Bacterium Paraburkholderia aromaticivorans AR20-38, a Gram-Negative, Cold-Adapted Degrader of Aromatic Compounds. Microbiol. Resour. Announc..

[36] Margesin R., Moertelmaier C., Mair J. (2013). Low-temperature biodegradation of petroleum hydrocarbons (n-alkanes, phenol, anthracene, pyrene) by four actinobacterial strains. Int. Biodeterior. Biodegrad..

[37] Allsop P.J., Chisti Y., Moo-Young M., Sullivan G.R. (1993). Dynamics of phenol degradation by *Pseudomonas putida*. Biotechnol. Bioeng..

[38] Nakazawa T., Nakazawa A. (1970). Pyrocatechase (Pseudomonas). Meth. Enzymol..

[39] Delgado-Baquerizo M., Oliverio A.M., Brewer T.E., Benavent-González A., Eldridge D.J., Bardgett R.D., Maestre F.T., Singh B.K., Fierer N. (2018). A global atlas of the dominant bacteria found in soil. Science.

[40] Männistö M.K., Häggblom M.M. (2006). Characterization of psychrotolerant heterotrophic bacteria from Finnish Lapland. Syst. Appl. Microbiol..

[41] Lapanje A., Wimmersberger C., Furrer G., Brunner I., Frey B. (2012). Pattern of elemental release during the granite dissolution can be changed by aerobic heterotrophic bacterial strains isolated from Damma Glacier (central Alps) deglaciated granite sand. Microb. Ecol..

[42] Berger T., Poyntner C., Margesin R. (2021). Culturable bacteria from an Alpine coniferous forest site: Biodegradation potential of organic polymers and pollutants. Folia Microbiol..

[43] Choo D.W., Kurihara T., Suzuki T., Soda K., Esaki N. (1998). A cold-adapted lipase of an Alaskan psychrotroph, *Pseudomonas* sp. strain B11-1: Gene cloning and enzyme purification and characterization. Appl. Environ. Microbiol..

[44] Tuorto S.J., Darias P., McGuinness L.R., Panikov N., Zhang T., Häggblom M.M., Kerkhof L.J. (2014). Bacterial genome replication at subzero temperatures in permafrost. ISME J..

[45] Margesin R., Bergauer P., Gander S. (2004). Degradation of phenol and toxicity of phenolic compounds: A comparison of cold-tolerant *Arthrobacter* sp. and mesophilic *Pseudomonas putida*. Extremophiles.

[46] Margesin R., Fonteyne P.-A., Redl B. (2005). Low-temperature biodegradation of high amounts of phenol by *Rhodococcus* spp. and basidiomycetous yeasts. Res. Microbiol..

[47] Kim D., Lee C.H., Choi J.N., Choi K.Y., Zylstra G.J., Kim E. (2010). Aromatic hydroxylation of indan by o-xylene-degrading *Rhodococcus* sp. strain DK17. Appl. Environ. Microbiol..

[48] Cloning and Characterization of Benzoate Catabolic Genes in the Gram-Positive Polychlorinated Biphenyl Degrader *Rhodococcus* sp. Strain RHA1|Journal of Bacteriology. https://journals.asm.org/doi/10.1128/JB.183.22.6598-6606.2001.

[49] Peng Y.-H., Chen Y.-J., Chang Y.-J., Shih Y. (2015). Biodegradation of bisphenol A with diverse microorganisms from river sediment. J. Hazard. Mater..

[50] Nweke C.O., Okpokwasili G.C. (2010). Influence of exposure time on phenol toxicity to refinery wastewater bacteria. JECE.

[51] Kasai D., Fujinami T., Abe T., Mase K., Katayama Y., Fukuda M., Masai E. (2009). Uncovering the Protocatechuate 2,3-Cleavage Pathway Genes. J. Bacteriol..

[52] Wang L., Nie Y., Tang Y.-Q., Song X.-M., Cao K., Sun L.-Z., Wang Z.-J., Wu X.-L. (2016). Diverse Bacteria with Lignin Degrading Potentials Isolated from Two Ranks of Coal. Front. Microbiol..

