# Supplementary material for: Biodegradation Potential and Putative Catabolic Genes of Culturable Bacteria from an Alpine Deciduous Forest Site
Source: Microorganisms. 2021 Sep 10;9(9):1920. doi: 10.3390/microorganisms9091920 (PMC8471709; doi:10.3390/microorganisms9091920)
Supplement: Supplementary file 1 [file microorganisms-09-01920-s001.zip › microorganisms-1363430-supplementary.pdf]

Supplementary Materials

Biodegradation potential and putative catabolic genes of culturable bacteria from an Alpine deciduous forest site

Caroline Poyntner[1], Andrea Kutzner[1] and Rosa Margesin[1]
[1]Department of Microbiology, University of Innsbruck, Technikerstrasse 25, 6020 Innsbruck, Austria

Table S1. Primers and PCR conditions for partial 16S rRNA gene amplification and the detection of putative catabolic genes: catechol-1,2-dioxygenase (C1,2D); multicomponent phenol hydroxylase (MPH); protocatechuate-3,4-dioxygenase (P3,4D).

Gene	Forward Primer 5'-3'	Reverse Primer 5'-3'	PCR conditons	Ref.
16S rRNA	AGAGTTTGATCCTGGCTC	AAGGAGGTGATCCAGCCGCA	i) 5 min at 95 °C;<br>ii) 35 cycles of 30 s at 95 °C, 30 s at 52 °C, 1 min at 72 °C;<br>iii) 10 min at 72 °C	[1]
C1,2D	CGCGGATTGTNGAYSTNTGGCANGCNAAYAC	GACTCAGGTNGCRWANGCRAARTCRTC	i) 3 min at 94 °C;<br>ii) 30 cycles 30 s at 94 °C, 40 s at 60 °C, 60 s at 72 °C;<br>iii) 10 min at 72 °C	[2]
MPH	GCGCGGAATTCGARTAYGTGYTGACCAA	GATCTCGAGCCGCGCCAGAACCAYTTRTC	i) 3 min at 94 °C;<br>ii) 30 cycles 30 s at 94 °C, 40 s at 60 °C, 60 s at 72 °C;<br>iii) 10 min at 72 °C	[2]
P3,4D	TGCCCNTACCACGGCTG	GCDCCGAKCTTCCAGTT	i) 5 min at 94 °C;<br>ii) 10 cycles 1 min at 94 °C, 1 min at 60 °C, 1 min at 72 °C;<br>iii) 15 cycles of 1 min at 94 °C, 1 min at 55 °C, 1 min at 72 °C;<br>iv) 15 cycles of 1 min at 94 °C, 1 min at 50 °C, 1 min at 72 °C;<br>v) 5 min at 72 °C	[3]

References

[1] Lane, D. 16S/23S rRNA sequencing. In *Nucleic acid techniques in bacterial systematics*, Stackebrandt, E., Goodfellow, M., Eds.; John Wiley & Sons: Chichester, 1991, pp. 115-175.

[2] Tuan, N.N.; Hsieh, H.-C.; Lin, Y.-W.; Huang, S.-L. Analysis of bacterial degradation pathways for long-chain alkylphenols involving phenol hydroxylase, alkylphenol monooxygenase and catechol dioxygenase genes. *Biores. Technol.* **2011**, *102*, 4232–4240.

[3] Li, C.; Zhang, C.; Song, G.; Liu, H.; Sheng, G.; Ding, Z.; Wang, Z.; Sun, Y.; Xu, Y.; Chen, J. Characterization of a protocatechuate catabolic gene cluster in *Rhodococcus ruber* OA1 Involved in naphthalene degradation. *Ann Microbiol* **2016**, *66*, 469–478.

Table S2. Identification of the 55 strains investigated, growth temperature range on R2A agar, effect of temperature (5°C and 20°C) on the utilization of organic compounds as sole carbon source, and presence of catabolic genes.

OD600:	< 0.100	0.1 - <0.25	0.25 - <0.5	0.5 - < 0.75	0.75 - >1	>=1

LSS, lignosulfonic acid; BPA, bisphenol A; C1,2D, catechol-1,2-dioxygenase; MPH, multicomponent phenol hydroxylase; P3,4D, protocatechuate-3,4-dioxygenase

Strain	Accession number	OTU	Phylum	Species	Growth temp. range (°C)	LSS				Catechol				Phenol				BPA				BPA + YE				C1,2D	MPH	P3,4D
						2 g/L 5°C	2 g/L 20°C	5 g/L 5°C	5 g/L 20°C	0.2 g/L 5°C	0.2 g/L 20°C	0.5 g/L 5°C	0.5 g/L 20°C	0.2 g/L 5°C	0.2 g/L 20°C	0.5 g/L 5°C	0.5 g/L 20°C	0.2 g/L 5°C	0.2 g/L 20°C	0.5 g/L 5°C	0.5 g/L 20°C	0.2 g/L 5°C	0.2 g/L 20°C	0.5 g/L 5°C	0.5 g/L 20°C			
AM0-02	MW969693	OTU17	*Actinobacteria*	*Rhodococcus sp.*	0-30	< 0.1	0.250	0.221	0.286	0.275	0.270	0.112	< 0.1	0.104	0.104	< 0.1	< 0.1	< 0.1	< 0.1	< 0.1	< 0.1	< 0.1	< 0.1	< 0.1	< 0.1			1
AM0-03	MW969733	OTU21	*Bacteroidetes*	*Pedobacter ginsenosidimutar*	0-30	< 0.1	< 0.1	< 0.1	< 0.1	< 0.1	0.120	0.133	0.322	< 0.1	< 0.1	< 0.1	< 0.1	< 0.1	< 0.1	< 0.1	< 0.1	< 0.1	< 0.1	< 0.1	< 0.1			
AM0-04	MW980058	OTU20	*Bacteroidetes*	*Duganella levis*	0-30	< 0.1	< 0.1	0.130	< 0.1	< 0.1	< 0.1	< 0.1	< 0.1	< 0.1	< 0.1	< 0.1	< 0.1	< 0.1	< 0.1	< 0.1	< 0.1	< 0.1	< 0.1	< 0.1	< 0.1			1
AM0-06	MW969759	OTU09	*Proteobacteria*	*Pseudomonas sp.*	0-30	0.136	< 0.1	0.299	0.141	0.231	0.112	< 0.1	0.105	0.241	0.220	0.105	0.270	< 0.1	< 0.1	< 0.1	< 0.1	< 0.1	0.152	< 0.1	0.237	1	1	1
AM0-07	MW969809	OTU04	*Proteobacteria*	*Collimonas sp.*	0-30	< 0.1	< 0.1	0.137	0.152	< 0.1	0.106	< 0.1	0.246	< 0.1	< 0.1	< 0.1	< 0.1	< 0.1	< 0.1	< 0.1	< 0.1	< 0.1	0.121	< 0.1	< 0.1			
AM0-10	MW969906	OTU19	*Proteobacteria*	*Pseudomonas jessenii*	0-30	< 0.1	< 0.1	0.104	0.111	< 0.1	0.135	< 0.1	0.201	< 0.1	< 0.1	< 0.1	< 0.1	< 0.1	< 0.1	< 0.1	< 0.1	< 0.1	< 0.1	< 0.1	0.109	1		1
AM0-14	MW971569	OTU18	*Proteobacteria*	*Serratia myotis*	0-35	< 0.1	0.735	0.118	0.475	< 0.1	< 0.1	< 0.1	0.104	< 0.1	0.483	< 0.1	0.108	< 0.1	0.259	< 0.1	< 0.1	< 0.1	< 0.1	< 0.1	< 0.1			
AM0-15	MW971570	OTU06	*Bacteroidetes*	*Pedobacter sp.*	0-30	< 0.1	< 0.1	< 0.1	< 0.1	< 0.1	< 0.1	< 0.1	0.137	< 0.1	< 0.1	< 0.1	< 0.1	< 0.1	< 0.1	< 0.1	< 0.1	< 0.1	< 0.1	< 0.1	< 0.1			
AM0-16	MW971571	OTU02	*Proteobacteria*	*Collimonas fungivorans*	0-30	< 0.1	< 0.1	< 0.1	< 0.1	< 0.1	< 0.1	< 0.1	0.110	< 0.1	< 0.1	< 0.1	< 0.1	< 0.1	< 0.1	< 0.1	< 0.1	< 0.1	< 0.1	< 0.1	< 0.1			
AM0-17	MW971573	OTU06	*Bacteroidetes*	*Pedobacter sp.*	0-30	< 0.1	< 0.1	< 0.1	< 0.1	< 0.1	< 0.1	< 0.1	0.174	< 0.1	< 0.1	< 0.1	< 0.1	< 0.1	< 0.1	< 0.1	< 0.1	< 0.1	< 0.1	< 0.1	< 0.1	1		1
AM0-18	MW971574	OTU02	*Proteobacteria*	*Collimonas fungivorans*	0-30	< 0.1	< 0.1	< 0.1	< 0.1	< 0.1	< 0.1	< 0.1	0.105	< 0.1	< 0.1	< 0.1	< 0.1	< 0.1	< 0.1	< 0.1	< 0.1	0.166	0.354	0.118	< 0.1	1		
AM0-19	MW971686	OTU11	*Proteobacteria*	*Serratia quinivorans*	0-35	0.220	0.681	0.302	0.752	< 0.1	< 0.1	< 0.1	< 0.1	0.145	0.365	< 0.1	0.137	< 0.1	0.681	< 0.1	< 0.1	< 0.1	< 0.1	< 0.1	< 0.1			
AM0-23	MW971687	OTU02	*Proteobacteria*	*Collimonas fungivorans*	0-30	0.138	< 0.1	0.163	< 0.1	< 0.1	< 0.1	< 0.1	< 0.1	< 0.1	< 0.1	< 0.1	< 0.1	< 0.1	< 0.1	< 0.1	< 0.1	< 0.1	< 0.1	< 0.1	< 0.1			
AM0-28	MW971904	OTU16	*Actinobacteria*	*Rhodococcus erythropolis*	0-35	0.417	1.107	0.426	0.945	0.329	0.592	< 0.1	0.255	0.340	0.842	0.303	0.947	< 0.1	< 0.1	< 0.1	< 0.1	< 0.1	< 0.1	< 0.1	< 0.1			
AM0-29	MW971928	OTU10	*Proteobacteria*	*Pseudomonas fluorescens*	0-30	< 0.1	< 0.1	< 0.1	< 0.1	< 0.1	< 0.1	< 0.1	0.133	< 0.1	< 0.1	< 0.1	< 0.1	< 0.1	< 0.1	< 0.1	< 0.1	< 0.1	< 0.1	< 0.1	< 0.1	1		1
AM0-30	MW979603	OTU02	*Proteobacteria*	*Collimonas fungivorans*	0-30	< 0.1	< 0.1	< 0.1	< 0.1	< 0.1	< 0.1	0.101	0.139	< 0.1	< 0.1	< 0.1	< 0.1	< 0.1	< 0.1	< 0.1	< 0.1	0.188	0.131	< 0.1	< 0.1			
AM0-31	MW979602	OTU13	*Proteobacteria*	*Glaciimonas immobilis*	0-25	0.391	0.291	0.376	0.248	< 0.1	< 0.1	< 0.1	< 0.1	0.275	0.451	< 0.1	0.297	< 0.1	< 0.1	< 0.1	< 0.1	< 0.1	< 0.1	< 0.1	< 0.1		1	
AM0-33	MW980053	OTU12	*Proteobacteria*	*Pseudomonas frederiksberge*	-5-30	< 0.1	< 0.1	0.180	0.139	< 0.1	< 0.1	0.106	0.141	< 0.1	< 0.1	< 0.1	< 0.1	< 0.1	< 0.1	< 0.1	< 0.1	< 0.1	< 0.1	< 0.1	< 0.1	1		1
AM0-34	MW979604	OTU08	*Firmicutes*	*Sporosarcina globispora*	-5-25	< 0.1	< 0.1	< 0.1	< 0.1	< 0.1	< 0.1	< 0.1	< 0.1	< 0.1	< 0.1	< 0.1	< 0.1	< 0.1	< 0.1	< 0.1	< 0.1	n. b.	n. b.	n. b.	n. b.			
AM0-35	MW979608	OTU01	*Proteobacteria*	*Pseudomonas sp.*	0-30	< 0.1	< 0.1	< 0.1	< 0.1	< 0.1	< 0.1	< 0.1	0.152	< 0.1	< 0.1	< 0.1	< 0.1	< 0.1	< 0.1	< 0.1	< 0.1	< 0.1	< 0.1	< 0.1	< 0.1	1		1
AM0-36	MW979609	OTU01	*Proteobacteria*	*Pseudomonas sp.*	0-30	< 0.1	< 0.1	< 0.1	0.158	< 0.1	< 0.1	< 0.1	0.108	< 0.1	< 0.1	< 0.1	< 0.1	< 0.1	< 0.1	< 0.1	< 0.1	< 0.1	< 0.1	< 0.1	< 0.1	1		
AM0-39	MW979611	OTU03	*Proteobacteria*	*Pseudomonas sp.*	-5-30	0.140	< 0.1	0.180	0.141	0.267	0.147	0.157	0.203	< 0.1	< 0.1	< 0.1	< 0.1	< 0.1	< 0.1	< 0.1	< 0.1	< 0.1	< 0.1	< 0.1	< 0.1	1		
AM0-44	MW979612	OTU03	*Proteobacteria*	*Pseudomonas sp.*	-5-30	< 0.1	< 0.1	< 0.1	0.135	< 0.1	0.130	< 0.1	0.137	< 0.1	< 0.1	< 0.1	< 0.1	< 0.1	< 0.1	< 0.1	< 0.1	< 0.1	< 0.1	< 0.1	< 0.1	1		1
AM0-51	MW979617	OTU01	*Proteobacteria*	*Pseudomonas sp.*	0-30	< 0.1	< 0.1	< 0.1	< 0.1	0.119	< 0.1	0.159	0.220	< 0.1	< 0.1	< 0.1	< 0.1	< 0.1	< 0.1	< 0.1	< 0.1	< 0.1	< 0.1	< 0.1	< 0.1	1		1
AM0-55	MW979619	OTU02	*Proteobacteria*	*Collimonas fungivorans*	0-30	< 0.1	< 0.1	0.105	0.138	< 0.1	0.101	< 0.1	0.130	< 0.1	< 0.1	< 0.1	< 0.1	< 0.1	< 0.1	< 0.1	< 0.1	n. b.	n. b.	n. b.	n. b.	1		
AM0-58	MW979618	OTU05	*Proteobacteria*	*Pseudomonas fluorescens*	0-30	< 0.1	0.115	0.117	0.184	0.274	0.168	< 0.1	0.221	< 0.1	< 0.1	< 0.1	< 0.1	< 0.1	< 0.1	< 0.1	< 0.1	< 0.1	< 0.1	< 0.1	< 0.1			
AM0-61	MW979620	OTU02	*Proteobacteria*	*Collimonas fungivorans*	0-30	< 0.1	< 0.1	0.140	0.128	< 0.1	0.108	< 0.1	0.201	< 0.1	< 0.1	< 0.1	< 0.1	< 0.1	< 0.1	< 0.1	< 0.1	n. b.	n. b.	n. b.	n. b.			
AM0-62	MW979691	OTU02	*Proteobacteria*	*Collimonas fungivorans*	0-30	< 0.1	< 0.1	0.119	0.129	< 0.1	0.117	< 0.1	0.203	< 0.1	< 0.1	< 0.1	< 0.1	< 0.1	< 0.1	< 0.1	< 0.1	0.162	< 0.1	0.111	< 0.1			
AM0-63	MW979751	OTU14	*Proteobacteria*	*Pseudomonas fluorescens*	0-30	< 0.1	< 0.1	< 0.1	0.226	0.356	0.269	0.115	0.100	< 0.1	< 0.1	< 0.1	< 0.1	< 0.1	< 0.1	< 0.1	< 0.1	< 0.1	< 0.1	0.112	< 0.1	1		
AM0-64	MW979752	OTU02	*Proteobacteria*	*Collimonas fungivorans*	0-30	< 0.1	< 0.1	0.133	0.130	< 0.1	0.113	< 0.1	0.218	< 0.1	< 0.1	< 0.1	< 0.1	< 0.1	< 0.1	< 0.1	< 0.1	< 0.1	< 0.1	0.307	< 0.1			
AM0-65	MW979753	OTU04	*Proteobacteria*	*Collimonas sp.*	0-30	< 0.1	< 0.1	0.109	0.117	< 0.1	0.118	0.103	0.251	< 0.1	< 0.1	< 0.1	< 0.1	< 0.1	< 0.1	< 0.1	< 0.1	< 0.1	< 0.1	< 0.1	< 0.1			
AM0-66	MW979755	OTU15	*Proteobacteria*	*Pseudomonas fluorescens*	-5-30	< 0.1	< 0.1	< 0.1	0.148	0.257	0.118	< 0.1	0.105	< 0.1	< 0.1	< 0.1	< 0.1	< 0.1	< 0.1	< 0.1	< 0.1	< 0.1	< 0.1	< 0.1	< 0.1	1		
AM0-68	MW979754	OTU23	*Firmicutes*	*Paenibacillus sinopodophylli*	0-30	< 0.1	< 0.1	< 0.1	0.134	< 0.1	0.112	< 0.1	0.215	< 0.1	< 0.1	< 0.1	< 0.1	< 0.1	< 0.1	< 0.1	< 0.1	< 0.1	< 0.1	< 0.1	< 0.1			
AM0-69	MW979756	OTU02	*Proteobacteria*	*Collimonas fungivorans*	0-30	< 0.1	< 0.1	< 0.1	0.129	< 0.1	0.116	< 0.1	0.213	< 0.1	< 0.1	< 0.1	< 0.1	< 0.1	< 0.1	< 0.1	< 0.1	< 0.1	< 0.1	< 0.1	< 0.1	1		
AM0-70	MW979766	OTU25	*Proteobacteria*	*Pseudomonas putida*	0-30	< 0.1	< 0.1	0.104	0.146	0.176	0.179	0.180	0.214	< 0.1	< 0.1	< 0.1	< 0.1	< 0.1	< 0.1	< 0.1	< 0.1	< 0.1	< 0.1	< 0.1	< 0.1	1		1
AM0-71	MW979814	OTU01	*Proteobacteria*	*Pseudomonas sp.*	0-30	< 0.1	< 0.1	< 0.1	< 0.1	< 0.1	0.135	0.124	0.152	< 0.1	< 0.1	< 0.1	< 0.1	< 0.1	< 0.1	< 0.1	< 0.1	< 0.1	< 0.1	< 0.1	0.306	1	1	1
AM0-72	MW979812	OTU01	*Proteobacteria*	*Pseudomonas sp.*	0-30	< 0.1	< 0.1	< 0.1	< 0.1	< 0.1	0.151	0.126	0.128	< 0.1	< 0.1	< 0.1	< 0.1	< 0.1	< 0.1	< 0.1	< 0.1	< 0.1	< 0.1	< 0.1	0.153	1	1	1
AM0-75	MW979813	OTU01	*Proteobacteria*	*Pseudomonas sp.*	0-30	< 0.1	< 0.1	< 0.1	< 0.1	< 0.1	0.129	0.117	0.119	< 0.1	< 0.1	< 0.1	< 0.1	< 0.1	< 0.1	< 0.1	< 0.1	< 0.1	< 0.1	< 0.1	0.351			1
AM0-76	MW979816	OTU01	*Proteobacteria*	*Pseudomonas sp.*	0-30	< 0.1	< 0.1	< 0.1	0.185	< 0.1	0.118	0.104	0.229	< 0.1	< 0.1	< 0.1	< 0.1	< 0.1	< 0.1	< 0.1	< 0.1	< 0.1	< 0.1	< 0.1	< 0.1			1
AM0-77	MW979817	OTU01	*Proteobacteria*	*Pseudomonas sp.*	0-30	< 0.1	< 0.1	< 0.1	< 0.1	< 0.1	0.151	0.118	0.111	< 0.1	< 0.1	< 0.1	< 0.1	< 0.1	< 0.1	< 0.1	< 0.1	< 0.1	< 0.1	< 0.1	0.306		1	1
AM0-81	MW979821	OTU02	*Proteobacteria*	*Collimonas fungivorans*	0-30	< 0.1	0.457	< 0.1	0.438	< 0.1	0.952	< 0.1	0.122	< 0.1	< 0.1	< 0.1	0.233	< 0.1	< 0.1	< 0.1	< 0.1	0.158	0.202	< 0.1	< 0.1			
AM0-82	MW979820	OTU01	*Proteobacteria*	*Pseudomonas sp.*	0-30	< 0.1	< 0.1	< 0.1	0.107	< 0.1	0.125	0.127	0.101	< 0.1	< 0.1	< 0.1	< 0.1	< 0.1	< 0.1	< 0.1	< 0.1	< 0.1	< 0.1	< 0.1	< 0.1		1	1
AM0-83	MW979866	OTU02	*Proteobacteria*	*Collimonas fungivorans*	0-30	< 0.1	< 0.1	< 0.1	< 0.1	< 0.1	< 0.1	< 0.1	< 0.1	< 0.1	< 0.1	< 0.1	< 0.1	< 0.1	< 0.1	< 0.1	< 0.1	< 0.1	< 0.1	< 0.1	< 0.1			1
AM0-84	MW979943	OTU22	*Bacteroidetes*	*Chryseobacterium solani*	-5-30	< 0.1	< 0.1	< 0.1	< 0.1	< 0.1	< 0.1	< 0.1	< 0.1	< 0.1	< 0.1	< 0.1	< 0.1	< 0.1	< 0.1	< 0.1	< 0.1	< 0.1	< 0.1	< 0.1	< 0.1			
AM0-85	MW979945	OTU01	*Proteobacteria*	*Pseudomonas sp.*	0-30	< 0.1	< 0.1	< 0.1	0.242	< 0.1	< 0.1	< 0.1	0.130	< 0.1	< 0.1	< 0.1	< 0.1	< 0.1	< 0.1	< 0.1	< 0.1	< 0.1	< 0.1	0.124	< 0.1			
AM0-87	MW979947	OTU24	*Bacteroidetes*	*Chryseobacterium polytrichas*	0-30	< 0.1	< 0.1	< 0.1	< 0.1	< 0.1	< 0.1	< 0.1	< 0.1	< 0.1	< 0.1	< 0.1	< 0.1	< 0.1	< 0.1	< 0.1	< 0.1	< 0.1	< 0.1	< 0.1	< 0.1			
AM0-89	MW979946	OTU01	*Proteobacteria*	*Pseudomonas sp.*	0-30	< 0.1	< 0.1	< 0.1	0.120	< 0.1	0.102	0.116	0.202	< 0.1	< 0.1	< 0.1	< 0.1	< 0.1	< 0.1	< 0.1	< 0.1	< 0.1	< 0.1	< 0.1	< 0.1	1		
AM0-90	MW979949	OTU03	*Proteobacteria*	*Pseudomonas sp.*	-5-30	1.006	0.981	1.032	1.003	1.231	0.933	0.421	0.833	0.505	0.529	0.171	1.267	0.339	1.033	0.230	1.498	< 0.1	< 0.1	< 0.1	< 0.1	1		1
AM0-92	MW979950	OTU03	*Proteobacteria*	*Pseudomonas sp.*	0-30	1.033	1.052	0.981	1.182	1.259	1.027	0.169	0.885	0.532	0.726	0.133	1.224	0.321	0.467	0.124	1.453	< 0.1	0.152	< 0.1	< 0.1	1		1
AM0-97	MW980002	OTU05	*Proteobacteria*	*Pseudomonas fluorescens*	-5-25	0.118	< 0.1	0.117	< 0.1	0.310	0.100	0.256	0.169	< 0.1	< 0.1	< 0.1	< 0.1	< 0.1	< 0.1	< 0.1	< 0.1	< 0.1	< 0.1	< 0.1	< 0.1			1
AM0-98	MW980001	OTU01	*Proteobacteria*	*Pseudomonas sp.*	-5-30	< 0.1	< 0.1	< 0.1	0.234	< 0.1	< 0.1	< 0.1	0.102	< 0.1	< 0.1	< 0.1	< 0.1	< 0.1	< 0.1	< 0.1	< 0.1	< 0.1	< 0.1	< 0.1	< 0.1	1		
AM0-99	MW980048	OTU02	*Proteobacteria*	*Collimonas fungivorans*	0-30	< 0.1	< 0.1	0.107	0.125	< 0.1	< 0.1	0.115	0.140	< 0.1	< 0.1	< 0.1	< 0.1	< 0.1	< 0.1	< 0.1	< 0.1	< 0.1	< 0.1	< 0.1	< 0.1	1		
AM0-101	MW981425	OTU01	*Proteobacteria*	*Pseudomonas sp.*	0-30	< 0.1	< 0.1	< 0.1	0.115	< 0.1	0.160	< 0.1	0.119	< 0.1	< 0.1	< 0.1	< 0.1	< 0.1	< 0.1	< 0.1	< 0.1	< 0.1	< 0.1	< 0.1	0.112			1
AM0-104	MW980052	OTU07	*Proteobacteria*	*Rahnella bruchi*	0-30	< 0.1	< 0.1	0.107	0.181	< 0.1	< 0.1	< 0.1	< 0.1	< 0.1	0.159	< 0.1	0.224	< 0.1	< 0.1	< 0.1	< 0.1	< 0.1	< 0.1	< 0.1	0.126	1	1	
AM0-106	MW980050	OTU07	*Proteobacteria*	*Rahnella bruchi*	0-30	< 0.1	< 0.1	0.133	0.193	< 0.1	< 0.1	< 0.1	0.101	0.112	0.154	< 0.1	0.160	< 0.1	< 0.1	< 0.1	< 0.1	< 0.1	< 0.1	< 0.1	0.104	1		

Table S3. Accession numbers of sequenced genes Catechol-1,2-dioxygenase (C1,2D), multicomponent phenol hydroxylase (MPH) and protocatechuate-3,4-dioxygenase (P3,4D) deposited at Genbank NCBI (https://www.ncbi.nlm.nih.gov/genbank/).

Strain	C1,2D	MPH	P3,4D
AM0-02			MZ603722
AM0-06	MZ574941	MZ617330	MZ603721
AM0-14			
AM0-19			
AM0-31		MZ617333	
AM0-39	MZ617341		
AM0-58			
AM0-63	MZ617335		
AM0-64			
AM0-66	MZ617336		
AM0-71	MZ617338	MZ617331	MZ603720
AM0-75			MZ617344
AM0-77		MZ617332	MZ617345
AM0-81			
AM0-85			
AM0-90	MZ617339		MZ617347
AM0-92	MZ617340		MZ617348
AM0-97			MZ617346
AM0-98	MZ617342		
